# Potential antiproteolytic effects of L-leucine: observations of *in vitro *and *in vivo *studies

**DOI:** 10.1186/1743-7075-5-20

**Published:** 2008-07-17

**Authors:** Nelo E Zanchi, Humberto Nicastro, Antonio H Lancha

**Affiliations:** 1 Laboratory of Applied Nutrition and Metabolism, Physical Education and School of Sports, University of São Paulo, São Paulo, Brazil

## Abstract

The purpose of present review is to describe the effect of leucine supplementation on skeletal muscle proteolysis suppression in both *in vivo *and *in vitro *studies. Most studies, using in vitro methodology, incubated skeletal muscles with leucine with different doses and the results suggests that there is a dose-dependent effect. The same responses can be observed in *in vivo *studies. Importantly, the leucine effects on skeletal muscle protein synthesis are not always connected to the inhibition of skeletal muscle proteolysis. As a matter of fact, high doses of leucine incubation can promote suppression of muscle proteolysis without additional effects on protein synthesis, and low leucine doses improve skeletal muscle protein ynthesis but have no effect on skeletal muscle proteolysis. These research findings may have an important clinical relevancy, because muscle loss in atrophic states would be reversed by specific leucine supplementation doses. Additionally, it has been clearly demonstrated that leucine administration suppresses skeletal muscle proteolysis in various catabolic states. Thus, if protein metabolism changes during different atrophic conditions, it is not surprising that the leucine dose-effect relationship must also change, according to atrophy or pathological state and catabolism magnitude. In conclusion, leucine has a potential role on attenuate skeletal muscle proteolysis. Future studies will help to sharpen the leucine efficacy on skeletal muscle protein degradation during several atrophic states.

## Introduction

Skeletal muscle atrophy is considered an important public health problem due to its primary (metabolic alterations) and secondary consequences (strength loss, decreased autonomy). As known, the atrophy process becomes apparent when skeletal muscle protein degradation is increased above protein synthesis during a prolonged period of time. In this context, leucine supplementation seems to be a promising anti-atrophy therapy, acting either by inhibiting skeletal muscle proteolysis and/or increasing protein synthesis, an effect which may possibly be both dose-dependent as well as skeletal muscle atrophy condition-dependent. This review will discuss the effects of leucine supplementation in the regulation of skeletal muscle proteolysis in both, in vitro and in vivo studies.

### Anti-atrophy effects of Leucine Supplementation

The sparing protein effects of leucine supplementation have been known since the initial studies of Buse & Reid (1975) [1]. Due to its properties of isolated action, leucine is actually considered not only an AA constituting protein, but also a physiopharmacological entity, whose administration is capable of promoting important anti-catabolic actions, such as attenuation of skeletal muscle catabolism during weight loss, facilitation of a healing process and improvement of skeletal muscle protein turnover in aged individuals [2,3].

In general, leucine supplementation consistently demonstrates decreases in skeletal muscle proteolysis when infused intravenously [4-8], incubated with the whole muscle [1,9-11], incubated with skeletal muscle cells [12,13] and under oral feeding [14]. It should be noted that the magnitude of inhibition is different according to the species, pathological condition and, especially, to the atrophy model. Importantly, in skeletal muscle cell cultures the antiproteolytic effects of leucine supplementation also occurs, but in this system the leucine dose-dependent effect appears to be shifted to a low concentration range when compared to the whole muscle incubation [15].

In chronic studies leucine supplementation efficacy is not as well demonstrated as in acute ones, but in this level leucine supplementation also shows important antiproteolytic effects. As a matter of fact, a recent study performed by Combaret *et al*. (2005) [14] showed that oral chronic leucine supplementation (~0.7 g/kg/day over a period of 10 days) presented long-lasting inhibitory effects on skeletal muscle proteolysis by around ~30%, restoring the defective postprandial inhibition of proteasome-dependent proteolysis. Also supporting the evidence of decreased proteolysis induced by leucine supplementation, a study performed by Ventrucci *et al*. (2004) [16] showed that in cancer cachexia-tumor induced rats, the consumption of an leucine enriched diet (3%) over a period of 20 days reduced protein degradation in gastrocnemius muscles by around 11%, increasing the myosin heavy chain content by around 47%. Recently, with the same study design, Eley *et al*. (2007) [17] demonstrated that the consumption of 1 g/kg/day of BCAA for 12–15 days suppresses the body weight loss by around 33% and decreases protein degradation by around 62% in soleus muscles. However, on the contrary, Sadiq *et al*. (2008) [18] showed that in calves, 5 days of intravenous infusion of EAA (rendering leucine plasmatic levels ~0.24 mM) under energy and protein deficits improved nitrogen balance, but the skeletal muscle proteolysis was not attenuated. Overall, chronic studies shown leucine supplementation as an important anti-atrophy strategy, but the lack of standardization related to the leucine supplementation dose (ingested and absorbed) impairs major conclusions related to the magnitude of effect. Table 1 summarizes important studies related to the leucine effects on skeletal muscle proteolysis in both humans and animals.

**Table 1:** Effects of leucine supplementation on animal e human studies related to skeletal muscle protein turnover

**Study**	**Duration**	**Dose**	**Methods**	**Results**
***Human***				

4	180 min	L-leucine: 1.09 and or 1.74 μmol/kg/min with insulin (plasma: 208 and 207 μmol/L) (infusion)	Leucine + KIC rates of appearance; KIC oxidation; Leucine-carbon flux	Leucine 1.09 μmol/kg/min stimulated leucine deposition into body protein in 37.7% but did not suppress endogenous proteolysis; Leucine 1.74 μmol/kg/min with insulin had a cumulative effect of 49.9% on net leucine deposition into body protein.
5	7 h	L-leucine: 154 ± 1 mmol/kg^-1^/h^-1 ^(infusion)	Whole-body valine and phenylalanine (tracers) flux	Inhibition of protein degradation without causing an increase in protein synthesis.
6	16 h	BCAA: 1.66 μmol/kg/min (infusion)	Whole-body (arterial and venous) leucine and phenylalanine (tracers) flux rates	Suppressed rate of whole-body (-37%) and forearm (-43%) muscle proteolysis.

***Rat***				

8^†^	105 min	BCAA: 246 mg/kg^-1^/h^-1 ^(infusion)	-	Neither muscle protein synthesis nor breakdown affected.
9	NR	L-leucine: 0.1, 0.2, 0.25 and/or 0.5 mM (muscle incubation)	Rate of ^14^CO_2 _and KIC production	Protein synthesis was stimulated in: 10% (0.1 mM), 19% (0.2 mM) and 42% (0.5 mM); Protein degradation was inhibited in: 0% (0.1 mM), 6% (0.2 mM), 15% (0.25 mM) and 26% (0.5 mM).
1	2 h	L-leucine: 0.5 mM (muscle incubation)	Incorporation of tyrosine (tracer) into proteins	Leucine increased the specific activity of the proteins by 25% and incorporated tissue proteins by 11.5%.
27	2 h	L-leucine: 0.5 mM (muscle incubation)	Rate of tyrosine (tracer) incorporated and released	Protein synthesis was stimulated in the soleus muscles by 69% and in EDL muscles by 38%. No effects on protein degradation were observed.
12	NR	L-leucine: 5 mM (muscle incubation)	Release of acid-soluble ^3^H-tyrosine (tracer)	Leucine caused a significantly reduction in proteolysis of -8 to 12%.
11	NR	L-leucine: 10 mM (muscle incubation)	Rate of tyrosine (tracer) released	Decreased whole-body proteolytic rate in 25%.
14	10 d	L-leucine: ~0,7 g/kg/day* (ingestion)	Rate of tyrosine (tracer) released	Suppressed postprandial proteolysis in old rats in 40% (measured by proteasome-dependent proteolysis).
17	12–15 d	BCAA: 1 g/kg/day* (ingestion)	Incorporation of L- [2,6-^3^H]phenylalanine and release of tyrosine (tracers)	Suppression on the loss of body weight (-1.5 vs. -4.5 g of control group); increase in rate of protein synthesis in gastrocnemius muscle (~50–60%) and in weight of the soleus muscle (~0.007 g); decrease of protein degradation in soleus muscle (-1500 g/2 h measured by fluorescence).

***Mice***				

16	20 d	L-leucine: ~4.2 g/day* (ingestion)	Rate of incorporation of [^3^H]-phenylalanine and release of tyrosine (tracers)	Protein synthesis was higher around 23.4% and degradation reduced in by around 11% with leucine supplementation.

***Calves***				

18	5 d	L-leucine: 239.6 μmol/L (infusion)	Urea creatinine, urea nitrogen and urea 3-methyl-histidine levels	Improvement on nitrogen balance without effect on protein degradation

^† ^= Unpublished results; *Relativized according to the data provided; AA = amino acid; BCAA = branched-chain amino acids; KIC = [1-14C]alpha-ketoisocaproate acod; NR = Not related.

### Acute Leucine Supplementation: Relationship between Dose and Effect

To bring about the efficacy of AA supplementation, it is necessary to understand its dose-effect properties. In this respect, Michael Rennie's group has made important discoveries related to the existence of a curvilinear dose-response relationship between the extra cellular EAA (particularly leucine among these) and the human skeletal muscle protein synthesis [19]. The concept is that under normal conditions, a single meal appears to promote maximum effects on protein synthesis. This finding is based on studies performed in both adult rats and adult humans, showing that the leucine plasma concentration in the PA state is approximately 0.1 mM [20,21] and in the PP state, it is increased approximately 80% or more, rendering mean values of 0.2 mM. This value seems to be capable of promoting maximum increases in the protein synthesis and of saturating the system [19,20].

When the issue is inhibition of protein degradation, much less is known about this property of dose-effect, even in animal studies. The results presented in the literature show variations, based on the skeletal muscle studied, the isotope utilized to measure protein metabolism and the system studied (*in vitro *vs *in vivo *studies). However, a deeper analysis of these studies, especially *in vitro studies *(where it is possible to simultaneously detect the protein synthesis and protein degradation metabolism), reveals new findings despite methodological differences. Thus, a re-interpretation of these studies is warranted to generate a new concept about a leucine dose-effect relationship.

In skeletal muscle cell cultures, the absence of aminoacids (especially leucine), appears to control protein degradation mainly through the activation of lysosome-dependent proteolysis [15,22] Additionally, during whole muscle incubations, leucine supplementation leads to a decreased ATP-ubiquitin-dependent proteolytic activity [11]. However, there is still no current mechanistic information about how the nutritional signal induced by AA is sensed by the muscle cell (i.e. intracellularly or extracellularly). Several studies by Mortimore and colleagues [23,24] and Pösö *et al*. (1982) [25], showed that in the liver, the extra cellular concentration of AA dictates in a dose-dependent fashion, the inhibition of proteolysis inside the liver cells, and that among AA, leucine was the strongest inhibitor.

When related to skeletal muscle, the intracellular AA concentration is not available in most studies. However, based on the extra cellular AA concentrations (plasmatic levels and/or extra cellular levels) it is possible to assume that the leucine concentration present to control the protein synthesis is also available to control the protein degradation, regardless of the mechanism process. Based on this assumption, the results of several studies are summarized below. They suggest that the leucine dose capable of causing maximal effects on protein degradation may be higher than that capable of causing maximal effects on protein synthesis during negative protein-balance conditions. However, this dose-dependent effect seems not occur in skeletal muscle cell cultures [15].

#### Animal Studies

In an earlier study in 1977, Buse & Weigand [26] showed that rat diaphragm muscles incubated with a leucine concentration of 0.5 mM (twice that of leucine concentration found in the PP state) was capable of increasing the skeletal muscle protein synthesis in 36–38%. Nevertheless, the protein degradation was inhibited in 4.7%, indicating that a high physiological leucine concentration is capable of both, strongly stimulating the protein synthesis and to a lesser extent, of inhibiting the protein degradation, even in the absence of a hormonal supply. In agreement with this, Tischler *et al*. (1982) [9], incubated rat diaphragm muscles with a wide leucine concentration (ranging from PA to PP state). They observed that a leucine concentration of 0.1 mM significantly increased the skeletal muscle protein synthesis. However, this same leucine concentration (0.1 mM) did not affect the rates of protein degradation, which were altered only when the leucine concentration was increased to a range from 0.2 to 0.5 mM. Within this range of concentration, protein degradation progressively diminished by a greater absolute amount than that which stimulated protein synthesis (Table 1).

In order to verify whether these results were observed only when AA were administered in vitro, Kee *et al*. (2003) [7] made a study using the extensor digitorum longus muscles, with the nutritional supply being provided during in vivo conditions (infused), in the presence of endogenous hormonal factors. The results showed that in 48 h starved rats, 4 h of AA infusion elevated the leucine plasma levels to values of approximately 0.57 mM. This increase on leucine plasma levels was capable of restoring the insulin and corticosterone levels to values observed in the controlled rats, concomitantly increasing the skeletal muscle protein synthesis in 55.6%. However, at the same leucine concentration, the skeletal muscle proteolysis was diminished to only 17.9% (value nonstatistically different from the starved group), suggesting that even in the presence of hormonal factors, the leucine concentration capable of stimulating the protein synthesis is attenuated when compared to protein degradation.

In another study, Hong & Layman (1984) [27] analyzed soleus muscles of starved rats (24 h and 72 h of fasting) incubated with leucine (0.5 mM). They observed that in the 24 h and 72 h fasted rats, protein synthesis was increased 59% and 24% respectively, but the protein degradation was not altered utilizing this leucine concentration in the soleus muscles. On the contrary, Busquets *et al*. (2000) [11], incubated rat soleus muscles with a higher leucine concentration of 5 mM and 10 mM. They observed that the skeletal muscle proteolysis was inhibited in a dose-response manner, i.e., leucine concentrations of 5 mM caused an inhibition of 5.7% in proteolysis, whereas 10 mM had caused an inhibition of 24.5%. This study reveals that increasing the leucine concentration to values 10–20× that of those observed in the other studies, was capable of bringing about decreases in skeletal muscle proteolysis, a result that is in accordance with another study conducted by Mitchell *et al*. (2004) [12] that observed that incubating skeletal muscle cells with a leucine concentration of 5 mM was capable of inhibiting skeletal muscle proteolysis in 8–12%. Thus, a plateau for a leucine concentration related to inhibition of skeletal muscle protein degradation during *in vitro *conditions was not yet established, but these studies suggest that a leucine concentration 10–20× superior to the one had in the PP state (~0.2 mM) is still capable of exerting its anti-proteolytic effects.

A limitation of the above studies was that although in vitro measurements *qualitatively *reflect rates of protein turnover that were present in the intact animal before the incubation period, the protein metabolism measured in the whole muscle during in vitro conditions was *always *in a pronounced state of negative protein balance even in control muscles [7]. However, this situation could mimic the one observed in an *in vivo *atrophic condition, where skeletal muscle protein degradation is increased over protein synthesis [28]. Thus, under certain atrophic conditions, it is possible that the amount of supplemented leucine capable of maximally inhibiting muscle proteolysis might be larger than that used to produce maximal effects on protein synthesis.

#### Human Studies

At the present, accurate methodologies have been utilized to measure protein synthesis in vivo but when the issue is protein degradation, different methods have presented important limitations, especially when related to protein degradation in muscle. For example, the urinary excretion of 3-methyl-histidine (3-MH) has been extensively used to estimate muscle protein breakdown, both in experimental animals and humans [29]. The rationale for using urinary 3-MH as a measure of skeletal muscle proteolysis is that the major portion of 3-MH is present in muscle actin and myosin and importantly, 3-MH is not reutilized for protein synthesis, being an index of protein degradation [30]. However, the specificity of urinary 3-MH excretion has been challenged, especially under in vivo conditions, because under some atrophic conditions like surgical trauma it was observed a disproportional overproduction of 3-MH from nonmuscle sources [31]. Another methodology adopted in vivo to measure protein degradation is to follow the loss of radioactivity from protein previously labeled by the administration of radioisotope tracer. When this method is used, a major concern is that radioactive AA derived from protein breakdown, enters the precursor pool and is reutilized for protein synthesis. Such recycling of labeled AA results in apparent breakdown rates which underestimate the true degradation rates [32]. Finally, an important question is the contribution of whole body versus skeletal muscle tissue in the protein degradation rates. To solve this question, several human studies [33,34] have utilized the arteriovenous net balance (NB) technique to evaluate rates of protein synthesis and protein breakdown in limbs (which are mostly muscle), and the amino acid phenylalanine has been used to trace muscle protein because it is neither produced nor metabolized in muscle. Using this technique, muscle protein breakdown can be estimated from the calculated value for the rate of phenylalanine appearance to the vein, during a steady state in the concentration of blood amino acids. However, a general problem with the use of either the forearm or leg arteriovenous NB technique to evaluate muscle metabolism acutely, is that this approach has practical limitations associated with the time and the blood required and, more importantly, does not permit the evaluation of short-lived effects on muscle protein metabolism [35], difficulting dose-response studies of proteolysis. At all, while there is a lack of reliable methods for measuring in vivo protein degradation in skeletal muscle, whole body protein breakdown can be estimated from the flux of radiolabeled or stable isotopes in plasma or nitrogen in urine.

Several authors have demonstrated that leucine as well as or BCAA supplementation (oral or infused) is capable of both, increasing protein synthesis [4,36,37] or decreasing protein degradation in humans [5,38,39]. There is a general tendency in most human studies to demonstrate that leucine supplementation is capable of promoting protein sparing effects, mostly due to inhibition of protein degradation [40]. Although the decline of proteolysis seemed to occur in several studies, there is no consensus of a dose-response relationship. For example, Tessari *et al*. (1987) [4] infused an AA solution in subjects (PA state) for 180 min, reaching leucine plasmatic levels of 0.2 mM, and no effects on endogenous proteolysis suppression were found, whereas Castellino *et al*. (1987) [41] infused an AA solution (subjects in the PA state) that rendered a leucine plasmatic concentration of 0.28 mM over a period of 180 min, and showed that the endogenous leucine flux (an indicator of proteolysis) was reduced 41.8% when compared to the basal period. These differences may be related to different methodologies used to analyze protein degradation, as suggested by Matthews (2005) [40] or even related to differential forms of leucine/AA administration in these studies, a limitation also observed in studies investigating skeletal muscle protein synthesis.

As stated above, there is no consensus about a dose-response relationship in human studies related to leucine supplementation and skeletal muscle proteolysis. However, a study performed by Sherwin (1978) [37] showed that obese subjects submitted to 3 days of fasting presented basal leucine levels almost doubled (0.22 mM) when compared to controlled subjects in the PA period, a leucine concentration normally capable of maximally stimulating the skeletal muscle protein synthesis in the PP period, under normal conditions. In this study, leucine infusion elevated leucine plasmatic concentration in 68% above the controlled subjects after two hours of infusion and 124% above the controlled subjects after 12 h (0.81 mM), under the same infusion rate. This acute leucine infusion (day 4 of fasting) was still capable of improving the nitrogen balance, returning to the previous levels on the day after the infusion. These results suggest that the *set point *of protein turnover metabolism related to the infused leucine concentration was up regulated in these subjects. In this study, the protein degradation was not inhibited by leucine infusion (measured by 3-methyl-histidine release), but the nitrogen balance was improved 23% after 12 h of leucine infusion. A recent study performed by Bohé *et al*. (2001) [42], indicated that in humans, infusing a solution composed of mixed AA in a rate that rendered a leucine plasmatic concentration of 0.4 mM, was capable of stimulating the protein synthesis for only two hours, returning to basal states after this period of time. Thus, it is possible that the inhibition of skeletal muscle degradation might also be contributed to the nitrogen sparing response during the 12 h of leucine infusion, at least in nonmuscular tissues.

Very recently, an interesting study made by Katsanos *et al*. (2006) [43], compared, in young and elderly subjects, the consumption of an oral solution composed of EAA containing a leucine concentration of 26% (which contained similar leucine values encountered in the whey protein) against the consumption of an EAA solution enriched with leucine 41%, on skeletal muscle protein metabolism. They observed that in young subjects, low dose leucine supplementation (26%) was capable to increase the skeletal muscle protein synthesis, whereas in the elderly subjects no effect was observed (although in both groups, similar plasmatic leucine levels were achieved ~0.45 mM). However, when a mixture containing high leucine concentration (41%) was supplemented, the elderly group increased the skeletal muscle protein synthesis to comparable values observed in young subjects, restoring the defective nutritional response observed in a low leucine supplemented dose (although again, in both groups, similar plasmatic leucine levels were achieved ~0.65 mM). Importantly, only the elderly subjects supplemented with the 41% enriched leucine solution presented a strong tendency to inhibit protein degradation. It is possible that in the elderly, a higher leucine supplementation dose will be capable to inhibit even more the muscle protein degradation, but this hypothesis was not yet tested.

Summing up, in human studies, leucine supplementation clearly induces an inhibition in skeletal muscle proteolysis and there are some sparse results suggesting that the leucine concentration capable of diminishing protein degradation may be larger than that capable of maximally stimulating the protein synthesis, especially under atrophic conditions.

## Summary and Conclusion

In the last few years, several studies have provided important advances on the understanding of how the feeding process is involved in the maintenance of skeletal muscle mass. After meal consumption, the feeding process provides in a coordinated fashion, rapid increases (~1–2 hour) in the skeletal muscle protein synthesis and inhibition of skeletal muscle protein degradation [44] that must be gradually reversed in the fasting period between each meal. The master nutritional signal involved in such responses appears to be the BCAA leucine [45]. However, after a prolonged atrophy state as that mediated by 48 h starvation in young rats, 4 h of AA infusion almost completely restores the protein synthesis process, whereas skeletal muscle proteolysis is not significantly affected [7]. Thus, under certain atrophic conditions, the nutritional signal provided by leucine does not appear to be simply the opposite way of the protein synthesis and protein degradation process. It is important to note that increasing the leucine concentration to values 10–20× superior (5–10 mM/L) to that capable of promoting maximal increases on protein synthesis, is still capable of exerting its skeletal muscle anti-proteolytic effects in a dose- dependent fashion [11]. Such evidences suggest that the nutritional dose-effect relationship provided by the leucine signal during certain atrophic conditions would be considered separately when related to skeletal muscle protein synthesis and skeletal muscle protein degradation. In agreement with this concept, during atrophic states as fasting and denervation, there are evidences that the protein degradation mechanisms are increased to an equal or even to a greater extension than that caused by the diminished protein synthetic mechanisms [46,47], but after a prolonged skeletal muscle inactivity period (28 days of bed rest with concomitant cortisone treatment), the atrophy process seems to be strictly related to the inhibition of protein synthesis [48]. Thus, if protein metabolism changes during different atrophic conditions, it is not surprising that the leucine dose-effect relationship must also change, as stated by Kobayashi and colleagues [8]. An important final consideration is related to the safety of leucine supplementation. BCAAs supplementation has minimal calories, does not stimulate gluconeogenesis, and does not increase the glomerular filtration rate that is reported to occur with alanine [2]. However, leucine is considered an important insulin secretagogue, and high/prolonged leucine supplementation is suggested to impair the glucose metabolism. As an example, an important study performed by Anello *et al*. (2001) [49], showed that chronic exposure of rat pancreatic islets to high (20 mM) leucine levels exhibited selectively impaired glucose-induced insulin release, but high leucine levels reported in the above study (1–10 mM) does not impaired the insulin secretion (although glucose skeletal muscle metabolism was not evaluated in this study). Future studies will help to sharpen the leucine efficacy and safety on skeletal muscle protein degradation during several atrophic states.

## Abbreviations

AA: Amino acid; BCAA: Branched chain amino acids; EAA: Essential amino acid; PA: Post absorptive; PP: Post prandial.

## Competing interests

The authors declare that they have no competing interests.

## Authors' contributions

NEZ, HN and AHL Jr conceived, drafted and approved the final manuscript.

## References

[B1] Buse MG, Reid SS (1975). Leucine: a possible regulator of protein turnover in muscle. J Clin Invest.

[B2] Tom A, Nair KS (2006). Assessment of branched-chain amino Acid status and potential for biomarkers. J Nutr.

[B3] Shimomura Y, Yamamoto Y, Bajotto G, Sato J, Murakami T, Shimomura N, Kobayashi H, Mawatari K (2006). Nutraceutical effects of branched-chain amino acids in skeletal muscle. J Nutr.

[B4] Tessari P, Inchiostro S, Biolo G, Trevisan R, Fantin G, Marescotti MC, Iori E, Tiengo A, Crepaldi G (1987). Differential effects of hyperinsulinemia and hyperaminoacidemia on leucine-carbon metabolism in vivo. J Clin Invest.

[B5] Nair KS, Schwartz RG, Welle S (1992). Leucine as a regulator of whole body and skeletal muscle protein metabolism in humans. Am J Physiol Endocrinol Metab.

[B6] Louard RJ, Barret EJ, Gelfand RA (1995). Overnight branched-chain amino acid infusion causes sustained suppression of muscle proteolysis. Metabolism.

[B7] Kee AJ, Combaret L, Tilignac T, Souweine B, Aurousseau E, Dalle M, Taillandier D, Attaix D (2003). Ubiquitin-proteasome-dependent muscle proteolysis responds slowly to insulin release and refeeding in starved rats. J Physiol.

[B8] Kobayashi H, Kato H, Hirabayashi Y, Murakami H, Suzuki H (2006). Modulations of muscle protein metabolism by branched-chain amino acids in normal and muscle-atrophying rats. J Nutr.

[B9] Tischler ME, Desautels M, Goldberg AL (1982). Does leucine, leucyl-tRNA, or some metabolite of leucine regulate protein synthesis and degradation in skeletal and cardiac muscle?. J Biol Chem.

[B10] Mitch WE, Clark AS (1984). Specificity of the effects of leucine and its metabolites on protein degradation in skeletal muscle. Biochem J.

[B11] Busquets S, Alvarez B, Llovera M, Agell N, López-Soriano FJ, Argilés JM (2000). Branched-chain amino acids inhibit proteolysis in rat skeletal muscle: mechanisms involved. J Cell Physiol.

[B12] Mitchell JC, Evenson AR, Tawa NE (2004). Leucine inhibits proteolysis by the mTOR kinase signaling pathway in skeletal muscle. J Surg Res.

[B13] Nakashima K, Ishida A, Yamazaki M, Abe H (2005). Leucine suppresses myofibrillar proteolysis by down-regulating ubiquitin-proteasome pathway in chick skeletal muscles. Bioch Biophys Res Commun.

[B14] Combaret L, Dardevet D, Rieu I, Pouch M, Béchet D, Taillander D, Grizard J, Attaix D (2005). A leucine-supplemented diet restores the defective postprandial inhibition of proteasome-dependent proteolysis in aged rat skeletal muscle. J Physiol.

[B15] Mordier S, Deval C, Béchet D, Tassa A, Ferrara M (2000). Leucine limitation induces autophagy and activation of lysosome-dependent proteolysis in C2C12 myotubes through a mammalian target of rapamycin-independent signaling pathway. J Biol Chem.

[B16] Ventrucci G, Mello MA, Gomes-Marcondes MC (2004). Proteasome activity is altered in skeletal muscle tissue of tumour-bearing rats a leucine-rich diet. Endocr Relat Cancer.

[B17] Eley HL, Russell ST, Tisdale MJ (2007). Effect of branched-chain amino acids on muscle atrophy in cancer cachexia. Biochem J.

[B18] Sadiq F, Crompton LA, Scaife JR, Lomax MA (2008). Effect of prolonged intravenous glucose and essential amino acid infusion on nitrogen balance, muscle protein degradation and ubiquitin-conjugating enzyme gene expression in calves. Nutr Metab (Lond).

[B19] Bohé J, Low A, Wolfe RR, Rennie MJ (2003). Human muscle protein synthesis is modulated by extracellular, not intramuscular amino acid availability: a dose-response study. J Physiol.

[B20] Dardevet D, Sornet C, Balage M, Grizard J (2000). Stimulation of in vitro rat muscle protein synthesis by leucine decreases with age. J Nutr.

[B21] Divino Filho JC, Bergstrom J, Stehle P, Forst P (1997). Simultaneous measurements of free amino acid patterns of plasma, muscle and erythrocytes in healthy human subjects. Clin Nutr.

[B22] Tassa A, Roux MP, Attaix D, Bechet DM (2003). Class III phosphoinositide 3-kinase – Beclin1 complex mediates the amino acid-dependent regulation of autophagy in C2C12 myotubes. Biochem J.

[B23] Mortimore GE, Khurana KK, Miotto G (1991). Amino acid control of proteolysis in perfused livers of synchronously fed rats. Mechanism and specificity of alanine co-regulation. J Biol Chem.

[B24] Mortimore GE, Wert JJ, Miotto G, Venerando R, Kadowaki M (1994). Leucine-specific binding of photoreactive Leu7-MAP to a high molecular weight protein on the plasma membrane of the isolated rat hepatocyte. Biochem Biophys Res Commun.

[B25] Pösö AR, Wert JJ, Mortimore GE (1982). Multifunctional control of amino acids of deprivation-induced proteolysis in liver. Role of leucine. J Biol Chem.

[B26] Buse MG, Weigand DA (1977). Studies concerning the specificity of the effect of leucine on the turnover of proteins in muscles of control and diabetic rats. Biochim Biophys Acta.

[B27] Hong SO, Layman DK (1984). Effects of leucine on in vitro protein synthesis and degradation in rat skeletal muscles. J Nutr.

[B28] Goldspink DF, Garlick PJ, McNurlanti MA (1983). Protein turnover measured in vivo and in vitro in muscles undergoing compensatory growth and subsequent denervation atrophy. Biochem J.

[B29] Hasselgren PO, Pedersen P, Sax HC, Warner BW, Fischer JE (1988). Methods for studying protein synthesis and degradation in liver and skeletal muscle. J Surg Res.

[B30] Wassner SJ, Schlitzer JL, Li JB (1980). A rapid, sensitive method for the determination of 3-methylhistidine levels in urine and plasma using high pressure liquid chromatography. Anal Biochem.

[B31] Rennie MJ, Bennegård K, Edén E, Emery PW, Lundholm K (1984). Urinary excretion and efflux from the leg of 3-methylhistidine before and after major surgical operation. Metabolism.

[B32] Swick RW, Ip MM (1974). Measurement of protein turnover in rat liver with (14C)carbonate. Protein turnover during liver regeneration. J Biol Chem.

[B33] Gelfand RA, Barrett EJ (1987). Effect of physiologic hyperinsulinemia on skeletal muscle protein synthesis and breakdown in man. J Clin Invest.

[B34] Tessari P, Barazzoni R, Zanetti M (1999). Differences in estimates of forearm protein synthesis between leucine and phenylalanine tracers following unbalanced amino acid infusion. Metabolism.

[B35] Katsanos CS, Chinkes DL, Sheffield-Moore M, Aarsland A, Kobayashi H, Wolfe RR (2005). Method for the determination of the arteriovenous muscle protein balance during non-steady-state blood and muscle amino acid concentrations. Am J Physiol Endocrinol Metab.

[B36] Giordano M, Castellino P, DeFronzo RA (1996). Differential responsiveness of protein synthesis and degradation to amino acid availability in humans. Diabetes.

[B37] Sherwin RS (1978). Effect of starvation on the turnover and metabolic response to leucine. J Clin Invest.

[B38] Ferrando AA, Williams BD, Stuart CA, Lane HW, Wolfe RR (1995). Oral branched-chain amino acids decrease whole-body proteolysis. J Parenter Enteral Nutr.

[B39] Hoffer LJ, Taveroff A, Robitaille L, Hamadeh MJ, Mamer OA (1997). Effects of leucine on whole body leucine, valine, and threonine metabolism in humans. Am J Physiol.

[B40] Matthews DE (2005). Observations of branched-chain amino acid administration in humans. J Nutr.

[B41] Castellino P, Luzi L, Simonson DC, Haymond M, DeFronzo RA (1987). Effect of insulin and plasma amino acid concentrations on leucine metabolism in man role of substrate availability on estimates of whole body protein synthesis. J Clin Invest.

[B42] Bohé J, Low JF, Wolfe RR, Rennie MJ (2001). Latency and duration of stimulation of human muscle protein synthesis during continuous infusion of amino acids. J Physiol.

[B43] Katsanos CS, Kobayashi H, Sheffield-Moore M, Aarsland A, Wolfe RR (2006). A high proportion of leucine is required for optimal stimulation of the rate of muscle protein synthesis by essential amino acids in the elderly. Am J Physiol Endocrinol Metab.

[B44] Yoshizawa F, Nagasawa T, Nishizawa N, Funabiki R (1997). Protein synthesis and degradation change rapidly in response to food intake in muscle of food-deprived mice. J Nutr.

[B45] Nagasawa T, Kido T, Yoshizawa F, Ito Y, Nishizawa N (2002). Rapid suppression of protein degradation in skeletal muscle after oral feeding of leucine in rats. J Nutr Biochem.

[B46] Pearlstein RA, Kohn RR (1966). Myosin and total protein turnover in denervated rat skeletal muscle. Am J Pathol.

[B47] Goldberg AL, Goodman HM (1969). Effects of disuse and denervation on amino acid transport by skeletal muscle. Am J Physiol.

[B48] Paddon-Jones D, Sheffield-Moore M, Cree MG, Hewlings SJ, Aarsland A, Wolfe RR, Ferrando AA (2006). Atrophy and impaired muscle protein synthesis during prolonged inactivity and stress. J Clin Endocrinol Metab.

[B49] Anello M, Ucciardello V, Piro S, Patané G, Frittitta L, Calabrese V, Giuffrida Stella AM, Vigneri R, Purrello F, Rabuazzo AM (2001). Chronic exposure to high leucine impairs glucose-induced insulin release by lowering the ATP-to-ADP ratio. Am J Physiol Endocrinol Metab.

